# The Innate Immune Protein Calprotectin Interacts With and Encases Biofilm Communities of *Pseudomonas aeruginosa* and *Staphylococcus aureus*

**DOI:** 10.3389/fcimb.2022.898796

**Published:** 2022-07-13

**Authors:** Jiwasmika Baishya, Jake A. Everett, Walter J. Chazin, Kendra P. Rumbaugh, Catherine A. Wakeman

**Affiliations:** ^1^ Department of Biological Sciences, Texas Tech University, Lubbock, TX, United States; ^2^ Department of Surgery, Texas Tech University Health Sciences Center, Lubbock, TX, United States; ^3^ Department of Biochemistry, Vanderbilt University, Nashville, TN, United States; ^4^ Department of Chemistry, Vanderbilt University, Nashville, TN, United States; ^5^ Center for Structural Biology, Vanderbilt University, Nashville, TN, United States; ^6^ Department of Immunology and Molecular Microbiology, Texas Tech University Health Sciences Center, Lubbock, TX, United States; ^7^ Texas Tech University Health Sciences Center Surgery Burn Center of Research Excellence, Texas Tech University Health Sciences Center, Lubbock, TX, United States

**Keywords:** calprotectin, *Pseudomonas aeruginosa*, *Staphylococcus aureus*, biofilm architecture, extracellular polymeric substance (EPS), nutritional immunity

## Abstract

Calprotectin is a transition metal chelating protein of the innate immune response known to exert nutritional immunity upon microbial infection. It is abundantly released during inflammation and is therefore found at sites occupied by pathogens such as *Pseudomonas aeruginosa* and *Staphylococcus aureus*. The metal limitation induced by this protein has previously been shown to mediate *P. aeruginosa* and *S. aureus* co-culture. In addition to the transition metal sequestration role of calprotectin, it has also been shown to have metal-independent antimicrobial activity *via* direct cell contact. Therefore, we sought to assess the impact of this protein on the biofilm architecture of *P. aeruginosa* and *S. aureus* in monomicrobial and polymicrobial culture. The experiments described in this report reveal novel aspects of calprotectin’s interaction with biofilm communities of *P. aeruginosa* and *S. aureus* discovered using scanning electron microscopy and confocal laser scanning microscopy. Our results indicate that calprotectin can interact with microbial cells by stimulating encapsulation in mesh-like structures. This physical interaction leads to compositional changes in the biofilm extracellular polymeric substance (EPS) in both *P. aeruginosa* and *S. aureus*.

## Introduction

Multiple pathogenic and commensal microbial species can colonize the human body. These microbial species often form highly adaptable, monomicrobial or polymicrobial communities called biofilms. Biofilms are encased in a matrix called the extracellular polymeric substance, or the EPS, that is typically produced by the microbes living within the biomass (Costerton et al., 1999). Microbial biofilms exhibit altered gene expression compared to their planktonic counterparts and have been shown to protect cells from environmental stresses such as antibiotics and phagocytosis (Römling and Balsalobre, 2012) (Stewart and Franklin, 2008; Flemming et al., 2016). The presence of the EPS as a physical barrier of drug penetration also provides antimicrobial resistance to biofilm-forming polymicrobial communities (Ren et al., 2019) and aids survival in harsh environmental conditions (Goltermann and Tolker-Nielsen, 2017). Additionally, the probability of exchange of DNA molecules containing antibiotic-resistance genes is also very high within cells in a biofilm (Águila-Arcos et al., 2017, Balcázar et al., 2015).

Most chronic infections such as cystic fibrosis (CF), chronic wound infections, periodontitis, chronic prostatitis, otitis media, *etc*. are associated with microbial biofilms (Donlan, 2002). According to the National Institutes of Health, biofilms account for almost 80% of all chronic microbial infections (James et al., 2008). *Pseudomonas aeruginosa* is a ubiquitous nosocomial pathogen particularly notable for the ability to form robust biofilms upon infection (Goltermann and Tolker-Nielsen, 2017).

To prevent colonization by pathogens, the host’s innate and adaptive immune responses possess various pattern-recognition receptors (PRRs), which detect microbial-associated molecular patterns (MAMPs) and trigger intracellular and intercellular signaling pathways (Medzhitov, 2007; Tan et al., 2018). The innate immune response is the first line of defense against bacterial infections. Its recognition of MAMPs is mediated by germline encoded PRRs, each of which can detect molecular structures unique to microorganisms (Janeway, 1989; Kubelkova and Macela, 2019). The PRRs have broad specificity and can often bind to many molecules with a common structural motif or pattern (Medzhitov, 2007). In addition to defending the host tissues *via* MAMP recognition-mediated phagocytosis, innate immune cells have other processes to inhibit invasion of tissues by pathogenic species. For example, host neutrophils have been shown to exert nutritional immunity on microbial pathogens such as *Pseudomonas aeruginosa* and *Staphylococcus aureus* (Corbin et al., 2008; Kehl-Fie et al., 2011; Zygiel et al., 2019). Nutritional immunity refers to withholding essential metals, such as iron, zinc, manganese, *etc*., from pathogens to hinder invasion and colonization (Hood and Skaar, 2012). Sequestration of transition metals is an effective antimicrobial strategy as these metals often serve as co-factors for essential enzymes in pathogens (Hood and Skaar, 2012; Ma et al., 2015). Transition metal-sequestering proteins are released from immune cells to bind free metal ions and decrease their concentration at infection sites. Calprotectin (CP) is one such metal-sequestering innate immune protein known for its ability to limit microbial access to multiple transition metal nutrients (Damo et al., 2013; Zygiel et al., 2019).

CP is a hetero-dimer of two S100 EF-hand Ca(II)-binding proteins, S100A8 (10.8 kDa) and S100A9 (13.2 kDa) that form an integrated structural unit with four EF-hand motifs. The CP heterodimer has two transition metal binding sites at the S100A8/S100A9 interface: a His3Asp motif (site 1) that chelates Zn(II) and Cu(II) and a His6 motif (site 2) that chelates these and other divalent metal ions, including Mn(II), Fe(II) and Ni(II) (Korndorfer et al., 2007; Damo et al., 2013; Nakashige et al., 2016; Gilston et al., 2016). The binding of Ca(II) or transition metals by CP promotes formation of a heterotetrameric dimer of dimers, and ultimately, higher order oligomeric states. All metal binding sites are energetically coupled, so binding of Ca(II) in the EF-hands increases the binding affinities at the transition metal site and vice versa (Obisesan et al., 2021).

In standard laboratory culture, *P. aeruginosa* is known to interact competitively with *S. aureus* (Mashburn et al., 2005; Beaume et al., 2015; Nguyen and Oglesby-Sherrouse, 2016). Typically, this competition results in *P. aeruginosa* dominating and outcompeting *S. aureus* growth. However, during infection, these two microbes are often found to coexist and exhibit a spectrum of microbial interactions ranging from competitive to cooperative depending on environmental context (Limoli and Hoffman, 2019). For example, CP exposure and zinc limitation has been shown to suppress competitive phenotypes in *P. aeruginosa* and permit coexistence with *S. aureus* (Wakeman et al., 2016; Vermilyea et al., 2021). Specifically, CP-mediated zinc limitation leads to repression of *P. aeruginosa* virulence factors such as pyocyanin and alkyl quinolones, which can promote co-infection with *S. aureus* in the murine lung (Wakeman et al., 2016). This suggests that the host immune protein CP can alter interactions between these pathogens.

In addition to the physiological effects of CP exposure mediated by transition metal sequestration, CP has been shown to interact with the surface of some pathogens to achieve an antimicrobial effect that is independent of metal starvation (Besold et al., 2018). Due to the ability of CP to impact bacterial cells in both a metal-dependent and contact-dependent manner, we sought to better understand the role that CP can play in the biofilm community structure of both mono- and polymicrobial communities of *S. aureus* and *P. aeruginosa*. We hypothesized that the surface interactions of CP associating to microbial cells could directly influence biofilm community extracellular polymeric substance (EPS) in a manner independent of zinc starvation. To test this hypothesis, we studied *S. aureus* and *P. aeruginosa* biofilm community structure in the presence and absence of CP using scanning electron microscopy (SEM) and confocal laser scanning microscopy (CLSM). Using these methods, we indeed observed direct interactions between CP and the cell surface of both Gram-positive and Gram-negative microbes and found that this interaction influenced biofilm EPS composition.

## Materials and Methods

### Bacterial Strains and Media

The *P. aeruginosa* strain used in the experiments is UCBPP-PA14, a highly virulent strain originally isolated from a wound infection (Rahme et al., 1995). The *S. aureus* strain used in the experiments is USA300 JE2, a laboratory-adapted strain derived from the parental strain, USA300, which was isolated from skin and soft tissue infection (Rahme et al., 1995). Both the strains were stored as glycerol stocks at -80°C and grown overnight at 37°C in Tryptic soy broth (TSB) for conducting any of the experiments described in this paper. The *P. aeruginosa* strain used in the *in vivo* infections is PAO1, a wild-type strain originally isolated from wound and is capable of producing exopolysaccharides such as Psl, Pel and alginate (Holloway, 1955).

### Chemicals and Reagents

All chemicals used to perform experiments were purchased from Sigma-Aldrich unless otherwise indicated. Wild-type human CP was expressed in *E. coli* and purified by ion exchange followed by size-exclusion chromatography as described previously (Kehl-Fie et al., 2011; Damo et al., 2013). Fixative solutions used to process biofilms for SEM and CLSM were purchased from Electron Microscopy Sciences (https://www.emsdiasum.com/microscopy/).

### Antibodies and Fluorescent Dyes

Human CP in culture media was detected by binding of the primary antibody, S100A8 Polyclonal Antibody (catalog number: PA5-82881, Invitrogen™) and mouse CP in chronic wound infections was detected by binding of the primary antibody, S100A8 Polyclonal Antibody (catalog number: BS-2696R, Invitrogen™). These were, in turn, bound by the fluorescently tagged secondary antibody, Goat anti-Rabbit IgG (H+L) Secondary Antibody, Alexa Fluor 488-10 nm colloidal gold (catalog number: A-31566, Invitrogen™). Microbial cell viability in *in vitro* samples was checked by staining biofilms with the cell-permeant dye, Hoechst 33342 Solution (20 mM) (catalog number: 62249, Thermo Scientific™). Microbial presence in *in vivo* samples was detected by labelling with the primary antibody, Anti-*Pseudomonas aeruginosa* antibody (catalog number: ab74980, abcam), coupled to a red-fluorescent secondary antibody, Goat Anti-Chicken IgY H&L (Alexa Fluor^®^ 594) preadsorbed (catalog number: ab150176, abcam). The biofilm EPS matrix components such as extracellular DNA (eDNA), matrix proteins, and carbohydrates such as α-mannopyranosyl and α-glucopyranosyl residues were detected by the fluorescent dyes, TOTO™-3 Iodide (642/660) (catalog number: T3604, Invitrogen™); FilmTracer™ SYPRO™ Ruby Biofilm Matrix Stain (catalog number: F10318, Invitrogen™); and Concanavalin A, Tetramethylrhodamine Conjugate (catalog number: C860, Invitrogen™), respectively. Fluorescently stained coverslips were mounted on glass slides using the ProLong™ Diamond Antifade Mountant (catalog number: P36961, Invitrogen™).

### *In Vitro* Co-Culture

Co-culture assays were performed in 50 mL conical tubes containing co-culture media (60% TSB; 40% calprotectin buffer [100 mM NaCl, 3 mM CaCl_2_, 10 mM β-mercaptoethanol, 20 mM Tris, pH 7.5)]. In the presence of CP and +zinc conditions, co-culture media was supplemented with 0.25 mg/mL WT calprotectin and 10 µM ZnCl_2,_ respectively. The 50 mL conical tube co-cultures were seeded with 1:100 dilutions of the metal-limited *P. aeruginosa* and/or *S. aureus* monocultures and grown for 42 hours statically at 37°C. The metal-limited mono-cultures *P. aeruginosa* and *S. aureus* used to seed the co-cultures were first grown overnight in glucose-supplemented low nutrient broth (GLNB) (2 g l−1 tryptic soy broth, 2 g l−1 glucose) at 37°C with shaking at 180 r.p.m. The next morning, cultures were metal-restricted by pelleting and suspending samples in Chelex 100-treated GLNB supplemented with 100 μM CaCl_2_ and 1 mM MgCl_2_. These cultures were grown at 37°C with shaking at 180 r.p.m. for 1.5 hours. Cultures were then pelleted, suspended in fresh metal-restricted GLNB, and grown for an additional 1.5 hours to produce metal-limited samples used for co-culture inoculation. Culture assays with Bovine serum albumin (BSA) were performed similarly in 50 mL conical tubes containing 0.25 mg/mL BSA added to TSB growth media.

### Murine Chronic Wound Infection Model

Adult female, non-diabetic Swiss Webster mice were anesthetized and depilated prior to administering full-thickness surgically excised wounds as previously described (Watters et al., 2013). To establish infection, 10^5^ CFU/mL of PAO1 was administered onto the wound bed. Wound-only groups were mock-infected with sterile phosphate-buffered saline. Uninjured mice were anesthetized, depilated, but not wounded. Mice were euthanized at 12-days post-injury and an approximately 2.5 cm^2^ area of the superficial murine dorsa, including wound bed and surrounding intact tissues, were resected for histology. Tissues were fixed with 10% neutral buffered formalin, embedded in paraffin and cut into sections.

### Scanning Electron Microscopy

For SEM, static cultures of *P. aeruginosa* and *S. aureus* were grown in 50 mL conical tubes with circular glass coverslips partially submerged in 1 mL of media. Coverslip samples were handled and processed as described previously (Gaddy et al., 2009). Briefly, cells on coverslips were fixed at room temperature for 1 hour using a primary fixative (2.5% glutaraldehyde, 2% paraformaldehyde solution in 0.05M sodium cacodylate buffer, pH 7.4). Fixed cells were then washed with 0.05 M sodium cacodylate to prevent sample dehydration. The coverslips were then incubated in 1% osmium tetroxide for 30 minutes, followed by a series of ethanol dehydration with increasing ethanol concentrations starting from 25% to 100 %. Finally, cells were dried using liquid CO_2_ at critical point temperature and pressure. Processed samples were gold-palladium coated before imaging using a Hitachi S-4300 scanning electron microscope.

### Confocal Laser Scanning Microscopy

For CLSM, *P. aeruginosa* and *S. aureus* static cultures were grown in 50 mL conical tubes with rectangular glass coverslips partially submerged in 3 mL of media. Post 42-hour incubation, coverslips were washed with phosphate-buffered saline to remove planktonic cells and fixed at room temperature for 1 hour using 4% paraformaldehyde solution. Fixed cells were washed with phosphate-buffered saline and blocked using 10% normal goat serum at room temperature for 1 hour. Blocked coverslips were incubated with S100A8 primary antibody and Goat anti-Rabbit Alexa Fluor 488 for 1 hour each to detect CP. To detect EPS matrix components, coverslips were incubated with either 2 µM TOTO-3 iodide for 20 minutes, or 200 µL of FilmTracer™ SYPRO™ Ruby Biofilm Matrix Stain for 30 minutes, or 200 µg/mL Concanavalin A, Tetramethylrhodamine Conjugate (TRITC Con A) for 1 hour. Coverslips were counterstained with the 20 µg/mL Hoechst 33342 for 15 minutes. Fluorescently labeled coverslips were mounted on glass slides and imaged at 100X magnification using the Olympus FV3000 Scanning Confocal Microscope.

### Confocal Imaging of Infected Mouse Wound Tissue

Tissue sections were de-paraffinized by washing them first with pure xylene twice (3 minutes each) and then with xylene mixed with 100% ethanol in a 1:1 ratio (3 minutes wash). Following this, tissue sections were treated twice with 100% ethanol and once each with 95%, 70% and 50% ethanol for 3 minutes each in the order mentioned and then submerged in cold tap water. Tris-EDTA buffer (10 mM Tris base, 1 mM EDTA solution, 0.05% Tween 20, pH 9.0) was used to perform heat-induced epitope retrieval on de-paraffinized sections. Briefly, the antigen retrieval buffer was heated to a temperature of ~98°C for 20 minutes on a hot plate. Tissue sections were placed inside 50 mL conical tubes containing the hot buffer solution and boiled for 20 minutes while being submerged within this solution. Post 20 minutes, tissue sections were rinsed with running cold tap water for 10 minutes and then blocked using 10% normal goat serum at room temperature for 1 hour. Blocked sections were incubated with the S100A8 and the Chicken anti-*P. aeruginosa* primary antibodies overnight at 4°C. The following morning, sections were incubated with the Goat anti-Rabbit Alexa Fluor 488 and the Goat Anti-Chicken IgY H&L (Alexa Fluor® 594) secondary antibodies simultaneously for 1 hour, each to detect CP and PAO1, respectively (Fleming et al., 2022). Labelled sections were finally counterstained with 2 μg/mL Hoechst 33342 for 15 minutes and imaged at 100X magnification using the Olympus FV3000 Scanning Confocal Microscope.

### Image Analyses

Image analyses were done using ImageJ for EPS quantification and CellSens for colocalization quantification. For EPS quantification, images were analyzed by splitting each image into RGB channels. Fluorescence integrated density values for TOTO-3 iodide/SYPRO Ruby/TRITC Con-A stains were collected from the red channel and for Hoechst 33342 stain from the blue channel. All fluorescence integrated density values were normalized based on the area and respective backgrounds. Average density values of each image were calculated and data was recorded as a ratio of TOTO-3 iodide/SYPRO Ruby/TRITC Con-A signals relative to Hoechst 33342 signals. Finally, the trimmed averages from each replicate (trimmed average represents the average of a dataset calculated by excluding the highest and lowest values of the set) were graphed using GraphPad Prism. For qualitative purposes, the brightness and contrast have been adjusted equally for all confocal images.

### Statistical Analyses

Statistical analyses were performed using GraphPad Prism 9.0 (GraphPad Software, Inc., San Diego, CA). Unpaired t-test (two-tailed) was used to calculate statistical significances.

## Results

### CP Leads to Encapsulation of *Pseudomonas aeruginosa* and *Staphylococcus aureus* in a Mesh-Like Structure

The interaction of pathogenic microorganisms with host immune molecules can play a significant role in their survival and colonization in human tissues (Baishya and Wakeman, 2019). Research conducted with CP and the causative agent of Lyme disease, *Borreliella (Borrelia) burgdorferi*, has shown that CP can inhibit the growth of *B. burgdorferi via* a mechanism that involves physical association of the protein with the bacteria (Besold et al., 2018). Additionally, exposure to CP has been shown to repress the production of anti-staphylococcal molecules within metal-deplete portions of *P. aeruginosa* biofilm and promote interaction between *P. aeruginosa* and *S. aureus* (Wakeman et al., 2016). Given these findings, we wanted to visualize the interaction of CP with *P. aeruginosa* and *S. aureus* biofilm communities in monoculture and co-culture using SEM.

**Figure 1:**
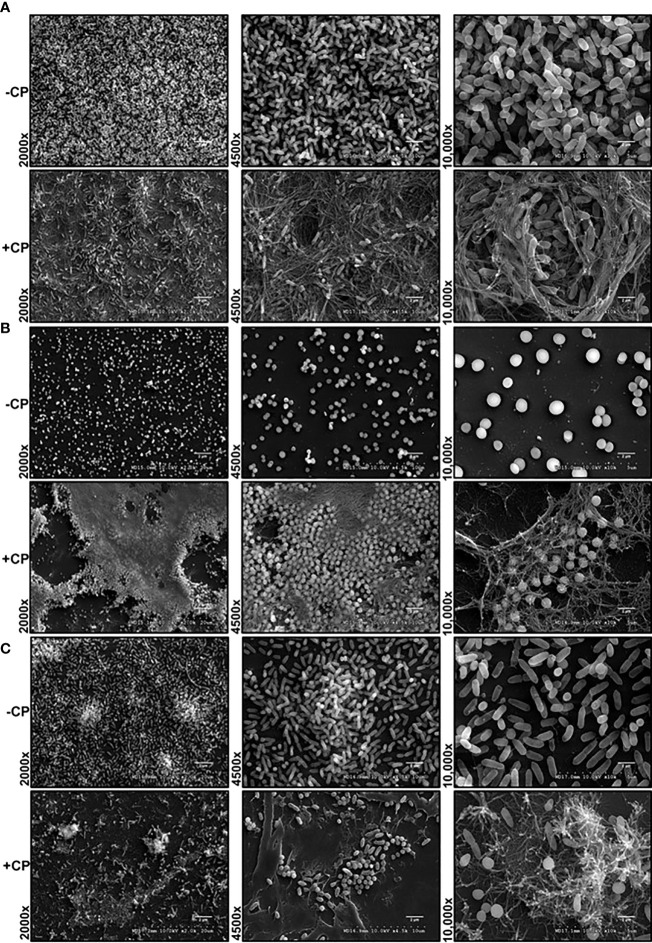
Addition of CP to growth media leads to encapsulation of *P. aeruginosa* and *S. aureus* in a mesh-like structure. SEM images of *P. aeruginosa* and *S. aureus* biofilms grown as mono and co-cultures in +/- CP conditions- **(A)** *P. aeruginosa* monoculture **(B)** *S. aureus* monoculture **(C)** *P. aeruginosa*-*S. aureus* co-culture. Images show 2000X, 4500X, and 10,000X magnification and are representative of three independent experiments.

Our SEM images of cultures grown in presence of 0.25 mg/mL CP, a physiologically relevant levels of this protein (Kehl-Fie et al., 2011), revealed the presence of a large and distinctive mesh-like structure that appeared to be encapsulating both *P. aeruginosa* and *S. aureus* cells (**Figure 1**). The structure was seen in both the monoculture conditions (**Figures 1A, B**) as well as the co-culture condition (**Figure 1C**). The observation that the formation of the mesh was not microbial species specific indicated that the enormous structure is likely not a bacterial EPS component but rather composed of an aggregation of the CP protein. However, to verify that the mesh formation in the presence of CP was not a cellular adaptation to CP-induced metal starvation, excess zinc was supplemented into the cultures to determine if the mesh-like structure was abolished by reversal of metal starvation. Since CP contains two metal binding sites, one known to be zinc/copper specific and one known to generally bind to multiple transition metals (Damo et al., 2013), saturation of these two sites with excess zinc is likely to generally alleviate CP-induced metal starvation. Supplementation with excess zinc did not reverse the formation of the mesh (**Supplementary Figures 1A–C**), indicating that the mesh is not simply an EPS adaptation to CP-induced metal restriction. Since cell surface contact between CP and microbial cells has previously been shown to be important (Besold et al., 2018), these mesh-like structures could be associated with the contact-based antimicrobial mechanism of CP.

Alternatively, these structures could represent a general aggregation phenotype of proteins added at high concentrations. To assess this possibility, we tested whether bovine serum albumin (BSA) will aggregate and cause a similar mesh structure at 0.25 mg/mL in the presence of biofilms. In this control experiment, the mesh structure was only observed in CP treated but not BSA treated biofilms (**Supplementary Figure 2**) suggesting that the structure observed in presence of CP may be unique to this protein.

### The Mesh-Like Structure Encasing Biofilms Contains Substantial Amounts of CP

**Figure 2:**
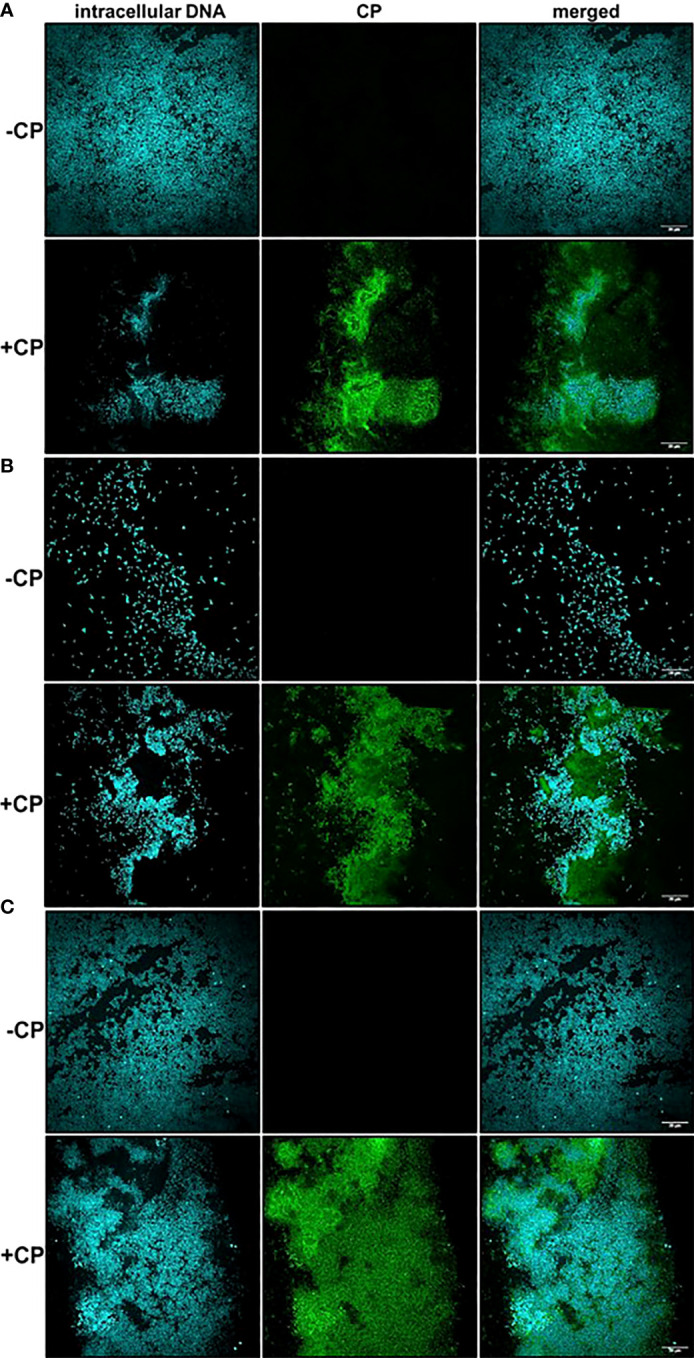
Calprotectin is a major component of the mesh-like structure that encapsulates *P. aeruginosa* and *S. aureus* biofilms. Intracellular DNA of microbial cells was detected by staining with the cell-permeant nucleic acid dye, Hoechst 33342; CP was detected by staining with Alexa fluor 488-tagged Goat anti-Rabbit antibody. **(A)** *P. aeruginosa* monoculture **(B)** *S. aureus* monoculture **(C)** *P. aeruginosa*-*S. aureus* co-culture. Images show 100X magnification and were processed using ImageJ.

After detecting the presence of a mesh-like structure in *P. aeruginosa* and/or *S. aureus* biofilm cultures containing the antimicrobial protein, CP, we wanted to confirm this structure contains CP. Therefore, we used immunofluorescence with a S100A8 Polyclonal primary antibody to directly detect CP presence in the biofilm structures. Our confocal images showed that in monocultures and co-culture of *P. aeruginosa* and/or *S. aureus*, only microbial cells were detected in the absence of CP, whereas both microbial cells as well as CP were detected in the presence of CP treatment (**Figures 2A–C**). Additionally, the signals denoting CP resembled the structure of the mesh seen in our SEM images where the CP-induced mesh appeared to be encapsulating *P. aeruginosa* and/or *S. aureus* cells within it. Thus, these results indicate that the mesh-like structure around microbial cells contains substantial amounts of CP and likely plays a role in CP’s interaction with microorganisms through a contact-based mechanism.

A striking and consistent observation from our CLSM images was that *S. aureus* monoculture biofilms grown in the absence of CP contained very few cells unlike the *P. aeruginosa* monoculture and the co-culture biofilms. Addition of CP led to an increase in *S. aureus* cells when grown as biofilms under static conditions at 37°C (**Figure 2B**). We were able to demonstrate that, while the growth conditions used in these studies did lead to a reduction in *P. aeruginosa* numbers, *S. aureus* viability was unaffected by our CP treatments (**Supplemental Figure 3**). Even though CP treatment was not toxic to *S. aureus* in our experimental conditions, it is surprising and interesting that a growth condition known to elicit zinc limitation would induce *S. aureus* biofilm formation given the fact that certain biofilm-associated proteins of *S. aureus* such as SasG have been shown to require zinc for biofilm formation (Formosa-Dague et al., 2016). Of note, it is known that *S. aureus* preferentially forms biofilms on substrates treated with host components such as human plasma (Chen et al., 2012; Cardile et al., 2014; Watters et al., 2016). These data indicate that, in *S. aureus* biofilms, CP-stimulated mesh-like structures might represent another host-derived substrate that can promote surface colonization, at least at sublethal concentrations of this protein.

### Accumulation of CP Around *P. aeruginosa* Occurs in Murine Chronic Wound Infections

**Figure 3:**
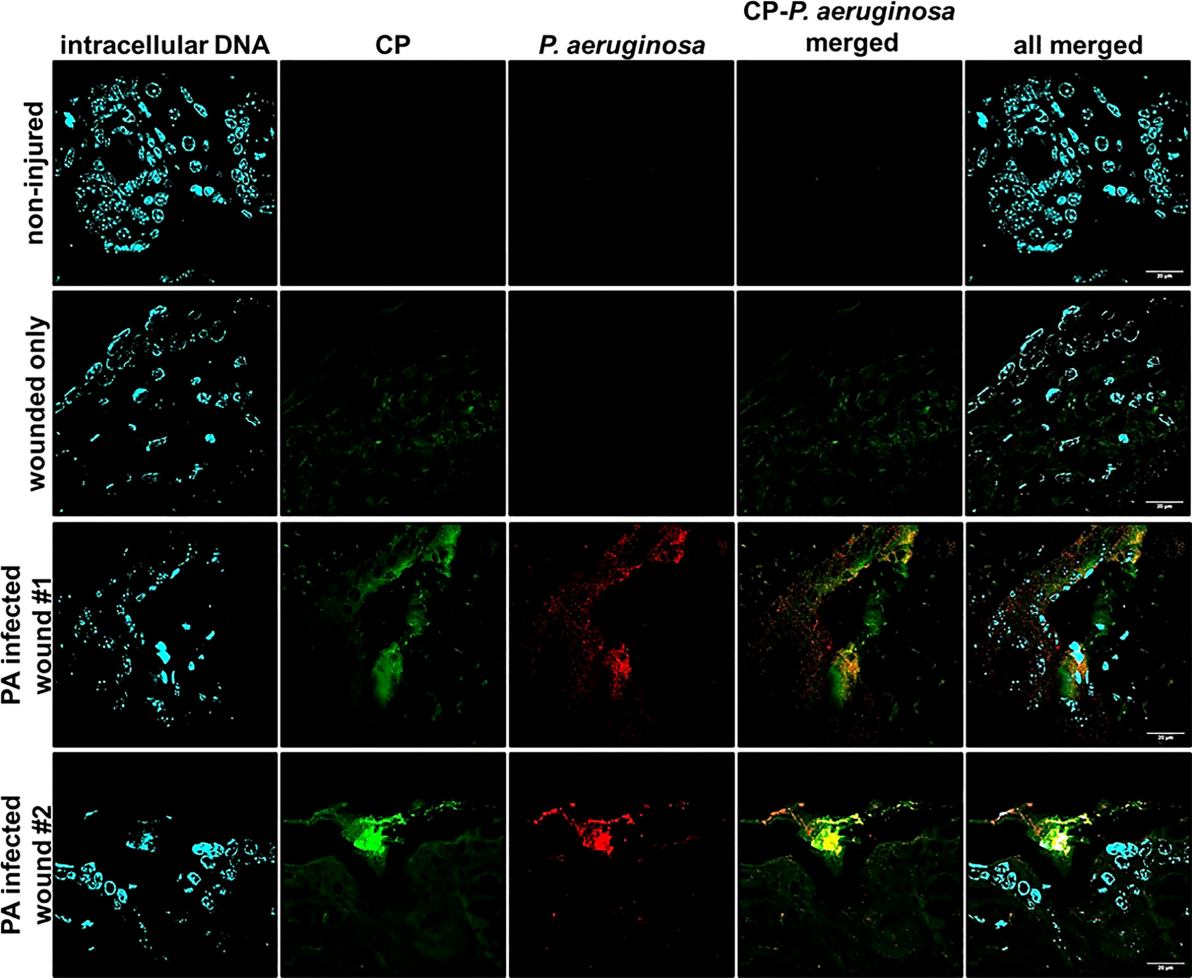
Mouse CP signals overlap with bacterial signals in paraffin-embedded dorsal tissue sections. Non-injured skin control collected 12 days post-depilation display no background CP or bacterial signal, 12-day uninfected wound only data reveals minimal CP signal and no background bacterial signal. Duplicate samples of 12-day PAO1-infected wounds reveal both CP and bacterial signals. CP was detected by staining with Alexa fluor 488-tagged Goat anti-Rabbit antibody, PAO1 was detected by staining with Alexa fluor 594-tagged Goat Anti- Chicken antibody and nuclei were detected by staining with the cell-permeant dye, Hoechst 33342. Images are shown at 100X magnification and processed using ImageJ.

To check if CP interacted with *P. aeruginosa* infecting host tissues *via* a similar mechanism involving mesh formation and encapsulation as seen under *in vitro* conditions, we stained PAO1-infected murine wound tissue sections using a mouse CP antibody and a *P. aeruginosa* staining antibody. The CLSM images from the prepared tissue sections from 12 day murine chronic wound infections revealed multiple areas consisting of an overlap of CP and PAO1 signals and an apparent encasement of PAO1 cells within CP-containing structures (**Figure 3**). The CP-stimulated structures detected in these more complex *in vivo* samples are not identical to the ones we showed in our *in vitro* samples (**Figure 2**), possibly due to the increased sample complexity or the additional processing required to remove the paraffin embedding matrix on the murine samples. However, the CP and *P. aeruginosa* aggregates in the *in vivo* samples do display enough similarities to our *in vitro* data to indicate that these findings might be relevant to structures occurring naturally during infection.

### The Carbohydrate Component of the EPS Is Enriched in the CP-Induced Matrix of *P. aeruginosa* and *S. aureus* Biofilms

**Figure 4:**
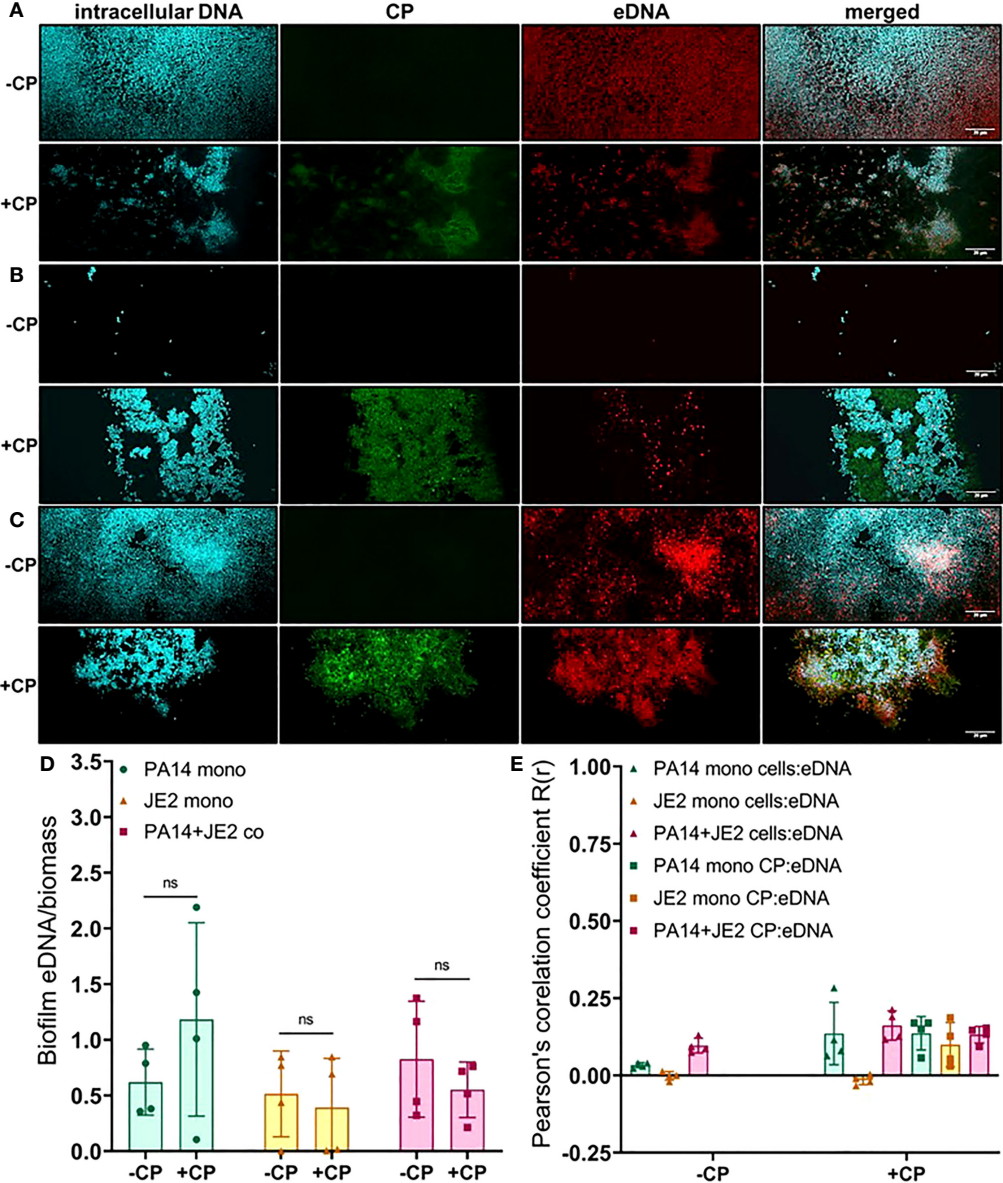
Addition of CP minimally impacts the relative abundance of eDNA in biofilm matrices of *P. aeruginosa* and *S. aureus.* This figure depicts confocal images that represent abundance of eDNA in *P. aeruginosa* and/or *S. aureus* biofilm communities in presence or absence of CP conditions. The fluorescence dyes Hoechst 33342, Alexa 488, and TOTO-3 iodide correspond to intracellular DNA of microbial cells, CP, and eDNA in the biofilm EPS matrix of- **(A)** *P. aeruginosa* monoculture **(B)** *S. aureus* monoculture **(C)** *P. aeruginosa*-*S. aureus* co-culture. **(D)** Quantification of eDNA per biomass in *P. aeruginosa* and *S. aureus* biofilms in presence or absence of CP. **(E)** Colocalization analysis of eDNA signals with cells and CP show minimal correlation of distribution of eDNA with cells and/or CP. Images show 100X magnification and were processed using ImageJ. Bars represent the mean of four biological replicates performed on two independent days. Error bars represent the standard error of mean of the biological replicates. Unpaired t-test (two-tailed) was used to measure statistical significance. Comparisons marked ns denote changes that were not found to be statistically significant.

As CP was directly interacting with microbial biofilms, we hypothesized that this contact might influence the production and localization of biofilm EPS components. To test this, we used fluorescent dyes to stain three major components of *P. aeruginosa* and *S. aureus* biofilm matrix, namely: eDNA; proteins; and carbohydrates. We first quantified the eDNA levels of the EPS in the presence or absence of CP. We hypothesized that the contact-mediated antimicrobial action of CP might result in increased eDNA release. Instead, we observed relatively small overall impacts on eDNA release relative to cell number in any of the conditions tested. If any trends could be inferred, it would be the opposite of what was hypothesized, with subtle trends towards a CP-mediated decrease in eDNA release per biomass in some cultures (**Figure 4**). Quantification of this data confirmed that no apparent trends were statistically significant (**Figure 4D**). We also compared the colocalization of eDNA in the biofilm matrix of both *P. aeruginosa* and *S. aureus* with cells and CP. Our results show no to minimal correlation with the distribution of eDNA signal with cell signal and/or CP signal in any tested conditions (**Figure 4E**).

**Figure 5:**
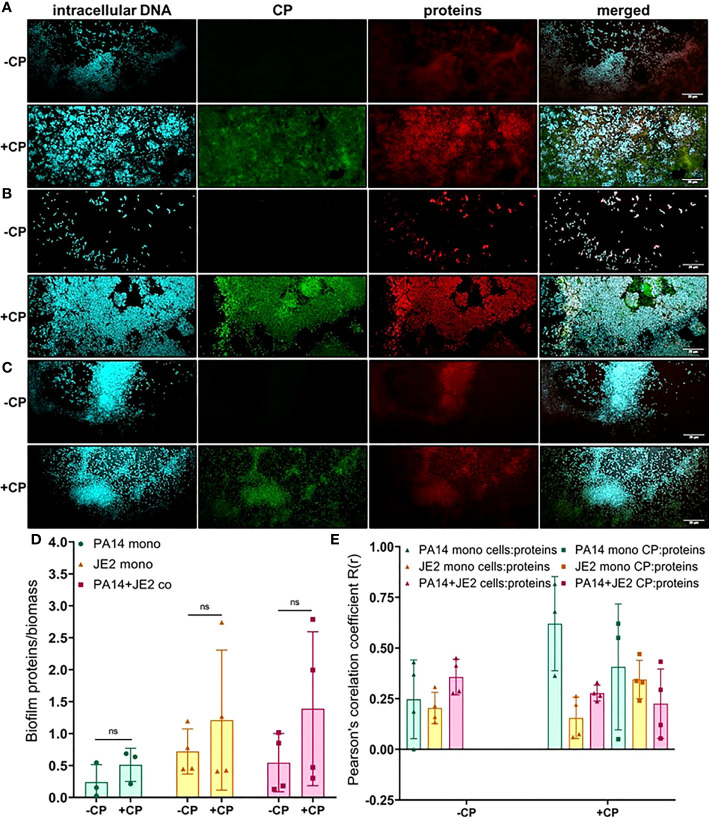
Addition of CP correlates with subtle increase in the protein component of biofilm matrices. This figure depicts confocal images that represent abundance of proteins in *P. aeruginosa* and/or *S. aureus* biofilm communities in presence or absence of CP conditions. The fluorescence dyes Hoechst 33342, Alexa 488, and SYPRO Ruby correspond to intracellular DNA of microbial cells, CP, and matrix proteins in the biofilm EPS of- **(A)** *P. aeruginosa* monoculture **(B)** *S. aureus* monoculture **(C)** *P. aeruginosa*-*S. aureus* co-culture. **(D)** Quantification of proteins per biomass in *P. aeruginosa* and *S. aureus* biofilms in presence or absence of CP. **(E)** Colocalization analysis of protein signals with cells and CP show similar distribution of protein signals around cell signals and CP signals. Images show 100X magnification and were processed using ImageJ. Bars represent the mean of four biological replicates performed on two independent days. Error bars represent the standard error of mean of the biological replicates. Unpaired t-test (two-tailed) was used to measure statistical significance. Comparisons marked ns denote changes that were not found to be statistically significant.

Next, we determined the impact of CP on protein component of the EPS using SYPRO ruby staining. These data indicate subtle trends in increased protein EPS component in cultures containing *P. aeruginosa* (**Figures 5A–C**). However, due to high variability in the data, these trends display no statistical significance (**Figure 5D**). Furthermore, this stain is a very general stain that can associate with CP. Therefore, it was especially important that we demonstrate that the SYPRO staining is not simply co-localizing with the CP signal. Some degree of co-localization of the CP signal and the general protein signal was observed, but this co-localization did not exceed what was observed for the correlation between the protein and cell signals (**Figure 5E**).

**Figure 6:**
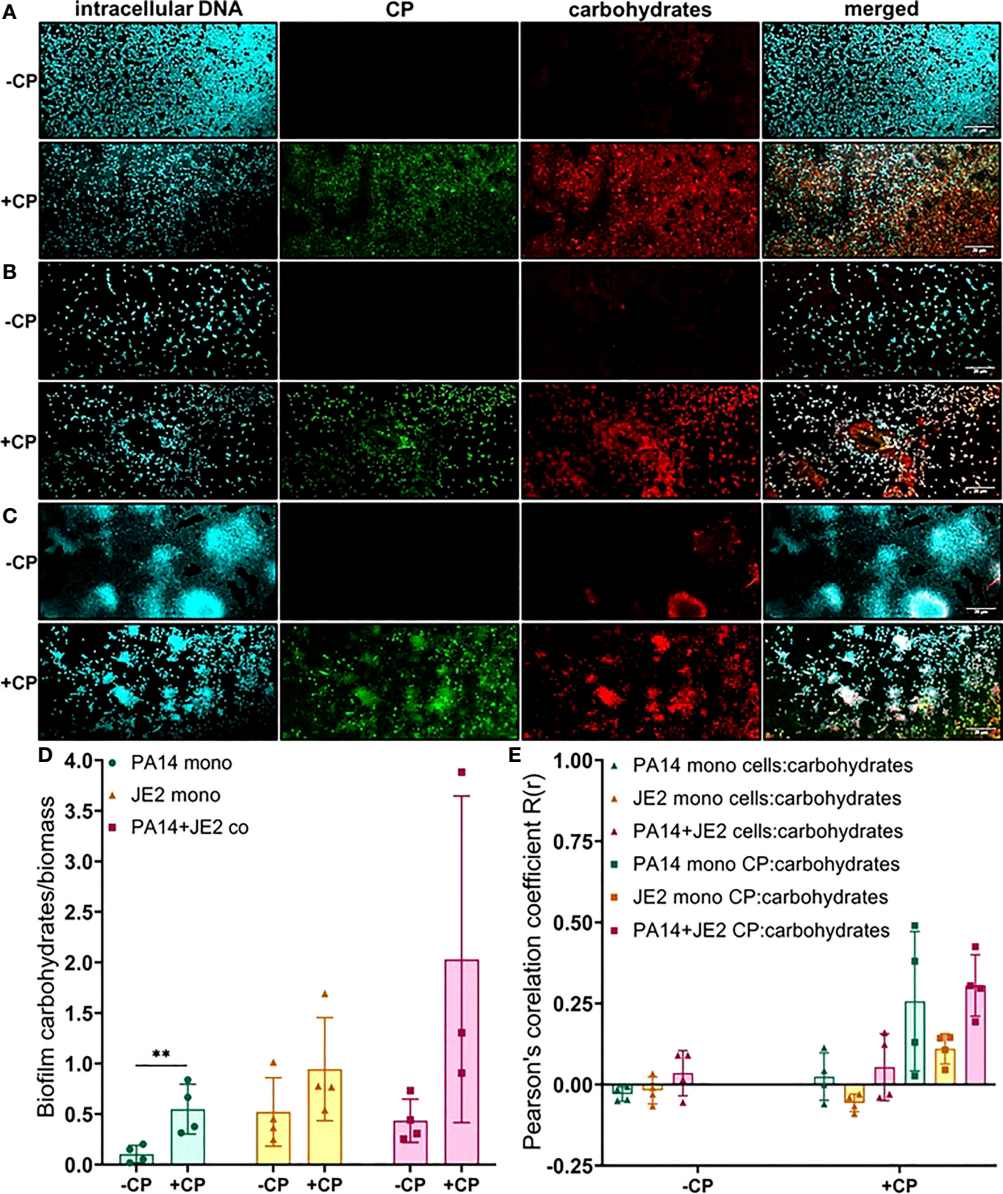
Addition of CP leads to an increase in EPS carbohydrates in *P. aeruginosa* monoculture biofilms. This figure depicts confocal images that represent abundance of carbohydrates in *P. aeruginosa* and/or *S. aureus* biofilm communities in presence or absence of CP conditions. The fluorescence dyes Hoechst 33342, Alexa 488, and TRITC ConA correspond to intracellular DNA of microbial cells, CP, and carbohydrates in the biofilm EPS of- **(A)** *P. aeruginosa* monoculture **(B)** *S. aureus* monoculture **(C)** *P. aeruginosa*-*S. aureus* co-culture. **(D)** Quantification of carbohydrates per biomass in *P. aeruginosa* and *S. aureus* biofilms in presence or absence of CP. **(E)** Colocalization analysis of carbohydrate signals with cells and CP signals show higher correlation with CP compared to cells. Images show 100X magnification and were processed using ImageJ. Bars represent the mean of four biological replicates performed on two independent days. Error bars represent the standard error of mean of the biological replicates. Unpaired t-test (two-tailed) was used to measure statistical significance. **p≤ 0.01.

Finally, we determined the impact of CP on carbohydrate molecules present in the biofilm EPS of *P. aeruginosa* and *S. aureus*. Our data indicate trends of CP-mediated increases of carbohydrate EPS components (specifically, α-mannopyranosyl and α-glucopyranosyl-containing ones based on the known staining preference of the concanavalin A dye) in all culture conditions (**Figure 6**); however, only the CP-induced increase seen in *P. aeruginosa* monoculture was determined to be statistically significant (**Figure 6D**). Colocalization data for carbohydrates in the biofilm matrix of both *P. aeruginosa* and *S. aureus* with cells and CP showed higher colocalization of CP with carbohydrates compared to cells for all culture conditions (**Figure 6E**), indicating a potential role of carbohydrate EPS molecules in combating CP-mediated antibacterial stresses. Overall, these data indicate that addition of CP can lead to alterations in the carbohydrate composition of *P. aeruginosa* and *S. aureus*’s biofilm matrix. These alterations are interesting and might be associated with microbial responses to the host environment.

## Discussion

Microorganisms are ubiquitous and exhibit physiological changes that allow them to adapt and survive in varying environments. These adaptations allow microorganisms to sustain life and multiply by utilizing the resources/factors unique to each environment. For example, biofilm communities of *P. aeruginosa* have been shown to exhibit temperature-specific adaptations in the human host versus in an industrial/environmental (i.e., soil/root associated or aquatic) surrounding (Bisht et al., 2021). Similarly, pathogenic microbial species rely on different biological processes when grown in laboratory conditions compared to when they infect and attempt to colonize human hosts (Cornforth et al., 2018; Ibberson and Whiteley, 2019). Colonization and survival in the human host environment can be different from lab conditions as it requires microbial species to counteract and overcome the host-associated antimicrobial molecules (Baishya and Wakeman, 2019). Therefore, studying the interactions between the human host immune components and pathogenic biofilm communities, at the host-pathogen interface, is a critical element for the discovery of unique and/or new targets for antimicrobial drugs and therapies.

CP is a human host innate immune protein that has been studied extensively for its metal sequestering properties (Kehl-Fie et al., 2011; Wakeman et al., 2016; Zygiel et al., 2019) and stimulation of inflammatory receptors including TLR4 and RAGE (Chen et al., 2013; Wang et al., 2018). CP is a heterodimer of the S100A8 and S100A9 proteins and is abundantly found within neutrophils, comprising approximately 50% of its cytoplasmic protein content (Edgeworth et al., 1991). In infected tissues, CP has been shown to sequester transition metals, such as zinc, from infecting pathogens to starve them, a phenomenon termed as nutritional immunity (Hood and Skaar, 2012). Since the role of CP is so critical to prevent microbial infections in humans, we wanted to explore the antimicrobial functions of CP in further detail, especially in the context of infections caused by the opportunistic and nosocomial pathogens, *P. aeruginosa* and *S. aureus*.

In this study, we have described experiments that were designed to investigate the interaction of CP with biofilm communities of *P. aeruginosa* and *S. aureus* using light microscopy (CLSM) as well as electron microscopy (SEM). Our SEM micrographs showed that presence of a mesh-like structure was seen around microbial cells grown in presence of CP (**Figure 1**). The mesh structure was confirmed to contain substantial amounts of CP by CLSM images where we detected strong fluorescence signals from CP-antibody complexes in the presence of CP cultures only (**Figure 2**). Additionally, accumulation of CP around *P. aeruginosa* was seen in CLSM images of mouse tissues infected by the pathogen (**Figure 3**). As CP is a metal chelating protein, we sought to investigate next if these mesh-like structures only form when the cells are starved for metals. However, we still observed the formation of this structure when excess exogenous ZnCl_2_ was added (**Supplementary Figure 1**). Given the extensive interaction of CP with the biofilm matrix, the fact that EPS composition changes in each biofilm type remained relatively subtle was surprising. Analysis of our confocal images exhibited a shift to a carbohydrate-rich composition of the EPS matrix in both *P. aeruginosa* and *S. aureus* (**Figure 6**). The carbohydrate component of the EPS seemed to preferentially co-localize with the CP signal as opposed to the cell-specific signal (**Figure 6E**). Finally, a general trend of increased ability for *S. aureus* to colonize non-human surfaces in the presence of the human protein CP was consistently observed in all CSLM data (**Figures 2B**, **4B**, **5B**, **6B**).The results from our experiments indicate that interaction of microbial species with the environment they are seeking to colonize play a significant role in the nature of the infection. As such, when colonizing the human hosts, interactions with the antimicrobial and immune components are crucial for infecting pathogens to be able to colonize the environment and establish an infection. Hence, exploring these interactions are crucial to the discovery and identification of key molecules and processes affecting the infecting microorganisms that may be useful for developing antibiotics. For example, our confocal results have shown that a shift in the EPS composition occurs in *P. aeruginosa* and *S. aureus* biofilms in presence of CP, an innate immune protein (**Figure 6**). This shift could be a result of adaptations by the microbial species in response to CP and can be, potentially, targeted by antibiotics and/or other therapeutics. In fact, targeting of *P. aeruginosa*’s EPS matrix components, specifically carbohydrates, by degrading enzymes such as glycoside hydrolases (GH) has recently been shown as an alternative to classical chronic infection treatments (Fleming and Rumbaugh, 2018; Redman et al., 2020; Kovach et al., 2020). EPS carbohydrates serve as the major EPS structural component in many pathogenic species and also play roles in the biofilm’s surface/cell-cell adhesion, aggregate formation, tolerance to desiccation, nutrient sorption, binding of enzymes and protection from antimicrobials and phagocytic cells (Flemming and Wingender, 2010; Yang et al., 2011).

*P. aeruginosa* produces three main types of carbohydrates/exopolysaccharides namely, Pel, Psl and alginate (Ma et al., 2009; Yang et al., 2011) and *S. aureus* mainly produces the poly N-acetyl glucosamine (PIA/PNAG) polysaccharide (Arciola et al., 2015). Active degradation of these EPS molecules *via* enzymes such as GHs could effectively break-up the pathogenic biofilms and disperse cells and in turn increase their susceptibility to antimicrobials and/or host immune cells (Fleming et al., 2017; Fleming and Rumbaugh, 2018). Thus, data from our research adds to this new pool of information that can lead to discovery of new and more effective methods of treating microbial infections with minimum off target effects.

While such alternative therapies are promising for treating biofilm-associated infections that are multi-drug resistant and recalcitrant, a large-scale dispersal of pathogenic cells within a living host can hyperactivate its immune system, causing dissemination of the infection and possibly lethal septicemia (Koo et al., 2017). Additionally, the biofilm composition of *P. aeruginosa* and *S. aureus* within the host-environment could differ significantly, and degrading enzymes would need to break up biofilms present in various physiologically distinct microenvironments that have differing nutritional profiles, oxygen levels, pHs, microbial community, etc. Hence, further studies exploring the biofilm composition of *P. aeruginosa* and/or *S. aureus in vivo* and the effect of CP on them is anticipated to enhance understanding of how host factors alter the communal biofilms in infected tissues and generate insights for the development of new antimicrobial therapeutics.

## Data Availability Statement

The raw data supporting the conclusions of this article will be made available by the authors, without undue reservation.

## Ethics Statement

The animal study was reviewed and approved by Institutional Animal Care and Use Committee of Texas Tech University Health Sciences Center (protocol number: 07044).

## Author Contributions

JB contributed to project design, data generation, data analysis, and manuscript writing. JAE performed the mouse infections and processed tissue samples. WC contributed to resource generation and manuscript editing. KPR contributed to design of the mouse infection experiments and manuscript editing. CW contributed to project design, data interpretation, and manuscript writing. All authors contributed to the article and approved the submitted version.

## Funding

Work in the Wakeman lab was supported by NIH/NIGMS (R15GM128072). Calprotectin research in the Chazin lab is supported by NIH/NIAID (R01 AI118089 and R01 AI127793). JB was supported by the Doctoral Dissertation Completion Fellowship granted from Texas Tech University Graduate School. JB received a publication award from Tech American Society for Microbiology (Tech ASM).

## Conflict of Interest

The authors declare that the research was conducted in the absence of any commercial or financial relationships that could be construed as a potential conflict of interest.

## Publisher’s Note

All claims expressed in this article are solely those of the authors and do not necessarily represent those of their affiliated organizations, or those of the publisher, the editors and the reviewers. Any product that may be evaluated in this article, or claim that may be made by its manufacturer, is not guaranteed or endorsed by the publisher.

## Supplementary Material

Supplementary Figure 1 Addition of zinc to CP-treated biofilm cultures of *P. aeruginosa* and *S. aureus* does not reverse and/or prevent the formation of the mesh. SEM images of biofilms grown in presence or absence of CP and +/-zinc conditions- **(A)**. *P. aeruginosa* monoculture **(B)**. *S. aureus* monoculture **(C)**. *P. aeruginosa*-*S. aureus* co-culture. Images show 4500X magnification and are representative of three independent experiments.

Supplementary Figure 2 Addition of equivalent concentration of BSA, a common host protein, to growth media does not lead to formation of the mesh structure that is seen around *P. aeruginosa* biofilms grown in presence of CP. SEM images of *P. aeruginosa* monoculture biofilms grown in presence of CP or BSA at 4500X magnification are shown. Due to a broken critical point dryer at the time these images were collected, the protocol for SEM sample processing was modified in the following manner, resulting in lower quality images: after standard biofilm growth on coverslips, cells were fixed at room temperature for 1 hour using 4% paraformaldehyde solution. Fixed samples were washed three times with distilled water and frozen as being submerged in distilled water at -80°C. Frozen samples were dehydrated via freeze drying overnight, then gold-palladium coated and subsequently image using a Hitachi S-4300 Scanning Electron Microscope.

Supplementary Figure 3 Cell viability following CP treatment in biofilm cultures of *P. aeruginosa* or *S. aureus.* Biofilms were grown in 0.25 mg/mL CP mimicking the growth conditions for the SEM and confocal cultures. These conditions resulted in a decrease in viability of *P. aeruginosa* but no change in *S. aureus* cell viability as determined by colony forming units (cfus). Data was generated on two independent days. Error bars represent standard deviation and * denotes statistical significance as determined by a two tailed Student’s t-test.
