# Supplementary material for: The Innate Immune Protein Calprotectin Interacts With and Encases Biofilm Communities of Pseudomonas aeruginosa and Staphylococcus aureus
Source: Front Cell Infect Microbiol. 2022 Jul 13;12:898796. doi: 10.3389/fcimb.2022.898796 (PMC9325956; doi:10.3389/fcimb.2022.898796)
Supplement: Supplementary Figure 1 — Addition of zinc to CP-treated biofilm cultures of P. aeruginosa and S. aureus does not reverse and/or prevent the formation of the mesh. SEM images of biofilms grown in presence or absence of CP and +/-zinc conditions- (A). P. aeruginosa monoculture (B). S. aureus monoculture (C). P. aeruginosa-S. aureus co-culture. Images show 4500X magnification and are representative of three independent experiments. [file Image_1.pdf]

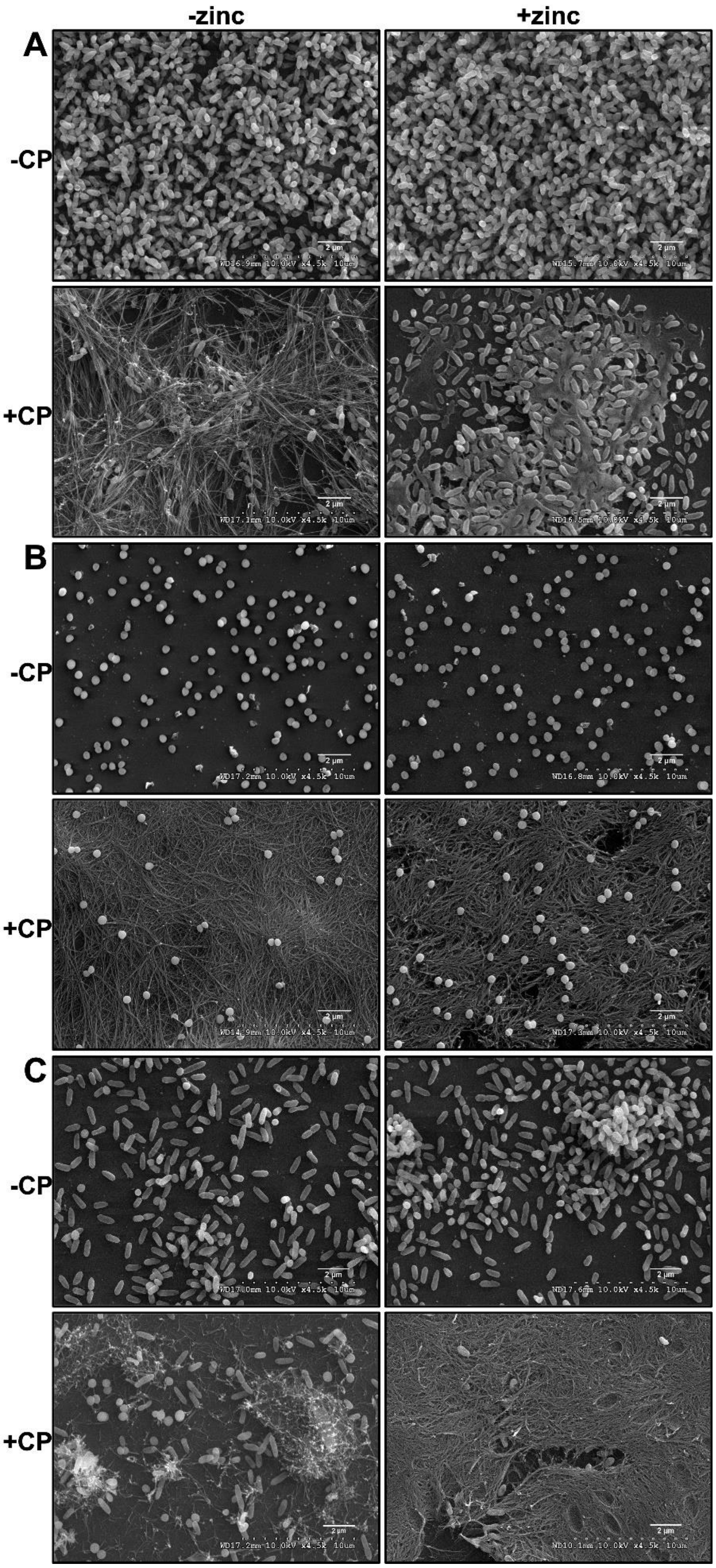

Supplementary Figure 1 | Addition of zinc to CP-treated biofilm cultures of *P. aeruginosa* and *S. aureus* does not reverse and/or prevent the formation of the mesh. SEM images of biofilms grown in presence or absence of CP and +/-zinc conditions (**A**). *P. aeruginosa* monoculture (**B**). *S. aureus* monoculture (**C**). *P. aeruginosa*-*S. aureus* co-culture. Images show 4500X magnification and are representative of three independent experiments.

Supplementary Figure 2 | Addition of equivalent concentration of BSA, a common host protein, to growth media does not lead to formation of the mesh structure that is seen around *P. aeruginosa* biofilms grown in presence of CP. SEM images of *P. aeruginosa* monoculture biofilms grown in presence of CP or BSA at 4500X magnification are shown. Due to a broken critical point dryer at the time these images were collected, the protocol for SEM sample processing was modified in the following manner, resulting in lower quality images: after standard biofilm growth on coverslips, cells were fixed at room temperature for 1 hour using 4% paraformaldehyde solution then washed three times with distilled water and frozen at -80°C submerged in distilled water. The samples were dehydrated with freeze drying overnight prior to gold-palladium coating and subsequent imaging.

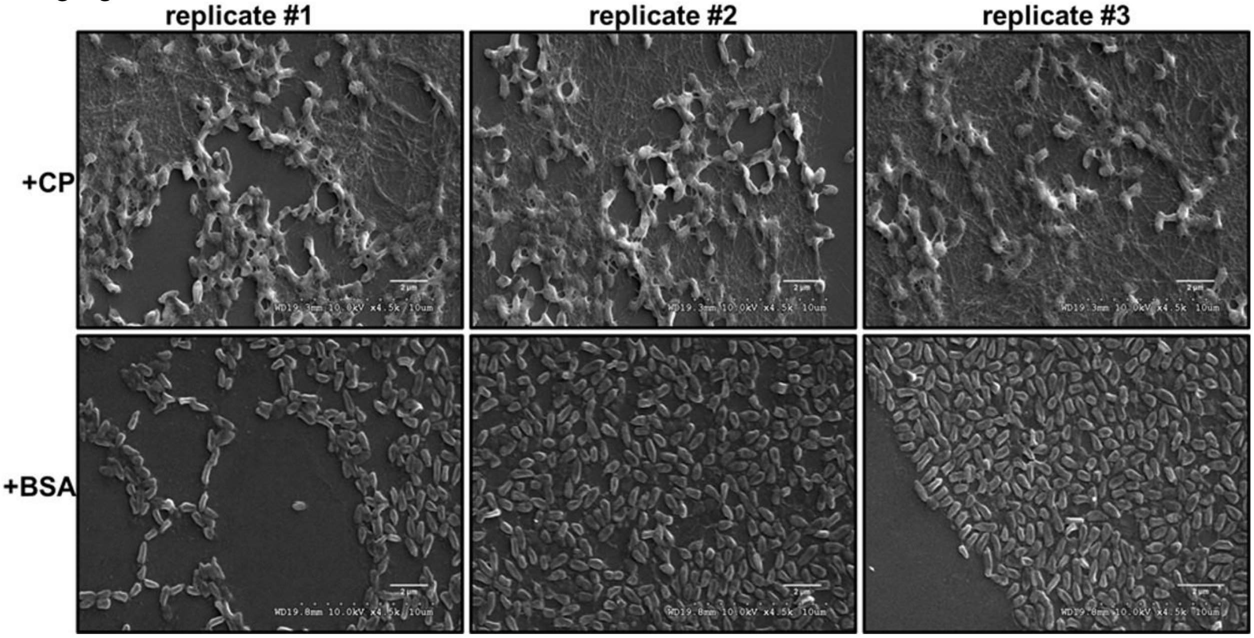

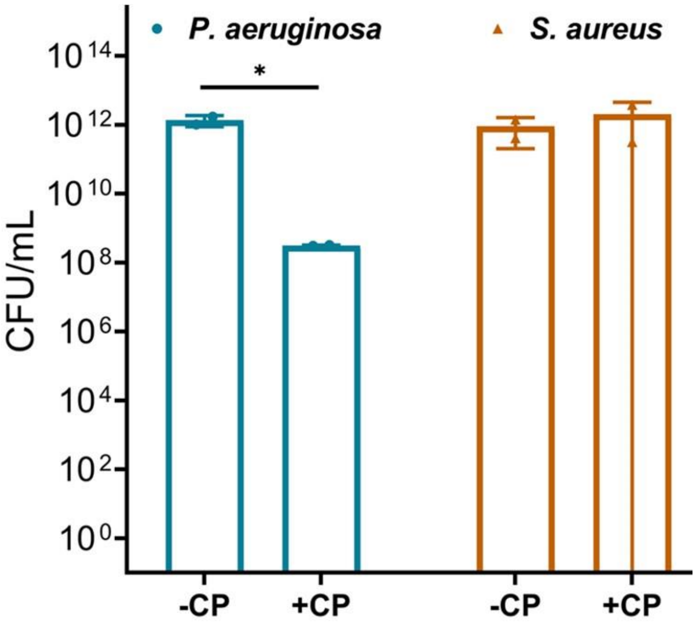

Supplementary Figure 3 | Cell viability following CP treatment in biofilm cultures of *P. aeruginosa* or *S. aureus*. Biofilms were grown in 0.25 mg/mL CP mimicking the growth conditions for the SEM and confocal cultures. These conditions resulted in a decrease in viability of *P. aeruginosa* but no change in *S. aureus* cell viability as determined by colony forming units (cfus). Data was generated on two independent days. Error bars represent standard deviation and * denotes statistical significance as determined by a two tailed Student's t-test
